# Chiral Growth of Gold Horns on Polyhedrons for SERS Identification of Enantiomers and Polarized Light-Induced Photothermal Sterilization

**DOI:** 10.3390/ma18112627

**Published:** 2025-06-04

**Authors:** Bowen Shang, Guijian Guan

**Affiliations:** Institute of Molecular Plus, Tianjin University, Tianjin 300072, China; 2022239030@tju.edu.cn

**Keywords:** material synthesis, chiral activity, surface-enhanced Raman scattering, enantiomer identification, circularly polarized light, bacterial inhibition

## Abstract

The integration of chirality into nanomaterials holds significant potential for improving molecular recognition and biomedical technologies. In this work, we fabricated novel chiral horned gold nanostructures (HNS) by controlling the concentration of chiral ligands L-/D-cysteine (Cys). The unique three-dimensional morphology with horns-rotational arrangement enables synergistic optimization of chiral optical responses and surface-enhanced Raman scattering (SERS) performance. The proposed chiral HNSs can be used to recognize amino acid enantiomers, in which homochiral amino acid has distinct affinities to the chiral HNSs of homogeneous handedness. The 4-mercaptobenzoic acid (4-MPBA)-modified D-HNS demonstrates significantly enhanced targeting affinity for D-amino acids in the *Escherichia coli* (*E. coli*) cell wall, enabling successful amplification of SERS signals and advancing bacterial detection methodologies. By demonstrating the rotation-selective interaction between chiral HNSs and circularly polarized light (CPL), D-HNS exhibits excellent photothermal conversion efficiency under right-handed circularly polarized light (RCP) irradiation. This enables the synergistic combination of targeted physical disruption and photothermal sterilization, which leads to efficient eradication of *E. coli*. The D-HNS hydrogel composite system further expands the practical application of photothermal sterilization. Altogether, chiral HNSs have achieved SERS detection of bacteria and efficient polarization photothermal sterilization, which helps further develop applications based on chiral nanomaterials.

## 1. Introduction

Chirality, a fundamental symmetry-breaking phenomenon in nature, manifests as molecules with identical functional groups displaying opposing optical and biological properties [1]. This intrinsic contrast drives the critical need for chiral substance identification, particularly in nanomaterial research and biological systems [2,3]. Conventional circular dichroism (CD) spectroscopy detects chirality through differential absorption of left-handed and right-handed circularly polarized (LCP and RCP) light, yet faces critical limitations: severe signal attenuation at trace concentrations, the inability to analyze molecular systems lacking characteristic chromophores, and exclusive reliance on electronic structure data from chiral centers–insufficient to distinguish structurally analogous enantiomers [4,5]. These inherent constraints underscore the urgent demand for developing direct, high-sensitivity chiral detection strategies.

Surface-enhanced Raman spectroscopy (SERS) exploits the localized surface plasmon resonance of noble metal nanostructures to detect molecular conformational features at the atomic vibration level, enabling unique identification of molecular fingerprints. This technique facilitates direct chiral molecular detection through the design of chiral plasmonic metamaterials capable of amplifying electromagnetic fields and chiral response signals [6,7,8]. Specifically, SERS substrates enhance enantiomer discrimination by leveraging localized electromagnetic field amplification and stereoselective adsorption, thereby achieving label-free in situ detection. Compared to conventional CD methods, SERS demonstrates superior sensitivity in differentiating enantiomers based on their functional group orientations (e.g., D-/L-amino acids) [9,10,11]. Additionally, the application potential of SERS in complex biological systems enables the detection and analysis of chiral molecules within microorganisms (e.g., identification of bacterial D-peptidoglycan), providing a feasible analytical approach for specific bacterial identification [12,13].

The development of stable chiral plasmonic substrates is critical for SERS-based enantiomer differentiation. Traditional plasmonic systems relying on symmetrical metallic nanostructures result in nearly identical adsorption configurations for enantiomers, limiting Raman signal divergence and hindering effective chiral discrimination. To overcome this challenge, plasmonic substrates must integrate intrinsic chirality, structural stability, and customizable surface functionalities that optimize both electromagnetic field enhancement and stereoselective interactions. Guided by this design strategy, chiral ligands such as amino acids and peptides have been employed to construct chiral plasmonic nanostructures through chiral transfer and molecular imprinting approaches [14,15,16], which can generate stereospecific binding sites and enhance enantioselective SERS responses.

Chiral plasmonic matrices have been extensively studied as antibacterial materials for drug-resistant bacteria [17,18]. However, the underlying mechanisms of chiral antibacterial activity remain poorly understood. Chiral nanostructures display distinct optical behaviors under circularly polarized light (CPL) [19,20], with their photothermal conversion properties dependent on CPL response. When the chirality of local surface plasmon resonance (LSPR) aligns with incident CPL, precise photothermal energy conversion is achievable [21,22]. This suggests that integrating chiral antibacterial effects with photothermal sterilization therapy could enhance antimicrobial efficacy.

Currently, chiral gold nanoparticles have achieved significant progress in synthetic strategies and applications [13,23], particularly in SERS-based enantiomer discrimination and photothermal sterilization. However, current studies still exhibit two critical limitations. First, most efforts focus on synthesizing chiral gold nanostructures with high g-factors to enable enantioselective SERS detection, yet lack systematic exploration of structure-oriented design. Traditional helical chiral nanostructures face challenges in trace enantiomer detection due to insufficient surface area and inhomogeneous plasmonic field distribution. Second, photothermal antibacterial systems predominantly rely on non-polarized light excitation, neglecting the unique polarization-dependent photothermal response of chiral nanostructures under CPL, which limits their chirality-driven antimicrobial potential. To address these limitations, we propose designing chiral gold nanoparticles with a multi-branched morphology, leveraging their high surface-to-volume ratios and enhanced LSPR-induced signal amplification to overcome the sensitivity limitations of SERS and enable trace enantiomer detection. Simultaneously, a synergistic photothermal sterilization platform combining CPL and chiral materials is introduced, where precise tuning of the polarization alignment between incident light and material chirality enables optimized antibacterial performance.

Conventional synthesis of chiral gold nanostructures often employs a seed-mediated approach combined with chiral ligand induction, successfully constructing typical morphologies such as propeller-like shapes [24], helical crystals [25], and screw-threaded nanorods [26]. The chirality origin in these structures primarily stems from the inherent structural distortion of the seed surface. However, transitioning from general chiral features to complex multi-branched chiral gold nanostructures remains a significant challenge, as the growth process lacks precise control over branching patterns and stable chiroptical signals. From a structural perspective, multi-branched chiral gold nanostructures require: (1) transitioning from traditional surface distortion to an epitaxial growth mode; (2) the seed surface providing enough growth-active sites; and (3) the multi-branched chirality manifesting as rotational arrangement with distinct tilt angles. To address these requirements, the multifaceted morphology of gold icosahedral seeds provides the prerequisites for multi-branched chiral gold nanostructures. When combined with high-concentration chiral ligand Cys, the synthesis of rotationally arranged branched structures is achieved. This constitutes a distinct difference from the inherent chiral structures formed by surface distortion in conventional chiral gold nanostructures.

In this study, gold polyhedrons were induced by chiral cysteine (Cys) to produce novel horned gold nanostructures (HNS) with chiral properties. The morphology and chiral optical signals of HNS can be altered by controlling the concentration of Cys, producing mirror-symmetric CD signals and tunable LSPR peaks. The optimized chiral HNSs demonstrated superior SERS performance, achieving threefold enantiomeric signal contrast between D- and L-phenylalanine (Phe), which was impossible with conventional gold nanoparticles. D-HNS has a stronger affinity for the D-amino acid-rich peptidoglycan layer for bacterial applications, thus binding preferentially to the bacterial cell wall. Functionalization with 4-mercaptophenylboronic acid (4-MPBA) enables ultrasensitive *E. coli* detection via enhanced SERS signals. In addition, under the irradiation of right-handed circularly polarized light (RCP), D-HNS exhibits polarization-selective photothermal conversion, which synergizes with physical destructive effects to achieve an efficient sterilization mechanism. This novel sterilization strategy, which integrates chiral nanoparticles with CPL, presents a promising approach for advancing research in antimicrobial technology.

## 2. Experimental Section

### 2.1. Chemicals

Tetrachloroauric acid (HAuCl_4_•3H_2_O, 99.9%), bovine serum albumin (BSA, 66 kDa), hydrogen peroxide (H_2_O_2_, 31 wt%), cetyltrimethylammonium bromide (CTAB, 99%), L-ascorbic acid (AA, 99%), 4-mercaptophenylboronic acid (4-MPBA, 95%), yeast extract (≥9%), agarose (99%), L-/D-/DL-cysteine (99%), L-/D-tryptophan (Trp, 99%), L-glutamate (Glu, 99%), L-penicillamine (Pen, 99%), and L-/D-phenylalanine (Phe, 99%) were purchased from Innochem. Sodium hydroxide (NaOH) and rhodamine 6G (R6G, 95%) were purchased from Aladdin and Biotopped, respectively. *E. coli* (OP50) was purchased from Bio Sci Biotechnology Co., Ltd., Hangzhou, China. All chemicals were used without further purification, and their aqueous solutions were prepared using ultrapure water.

### 2.2. Synthesis of Chiral and Achiral HNSs

The growth/formation of gold horns was realized by a seed-mediated approach. Typically, the gold polyhedron seeds were synthesized via the destabilization and regrowth of gold nanoclusters after adding H_2_O_2_, similar to our previous literature [27]. In the experiment, a mixed solution containing 2.5 mM HAuCl_4_ and 15 mg/mL BSA was prepared via vigorous stirring for 10 min, and then a certain amount of NaOH solution (1 M) was injected to form a pH = 11 condition. The reaction temperature was heated to 40 °C and maintained at it for 12 h to produce a brown solution of BSA-protected Au_25_ nanoclusters. After cooling to room temperature, 4 mL of the prepared Au_25_ solution was added to a glass vial and 500 mM H_2_O_2_ was introduced. The sealed glass vial was shaken for 30 s and then allowed to stand at room temperature for 72 h. With the change in the solution from brown to colorless to red, the gold polyhedrons were synthesized from Au_25_ nanoclusters. The final solution was washed three times by centrifugation with distilled water and redispersed in 4 mL of distilled water for further use as the seed solution.

To synthesize chiral and achiral HNSs, the growth solution was first prepared by thoroughly mixing 0.8 mL of CTAB (100 mM) and 0.2 mL of HAuCl_4_ (10 mM) into 4 mL of ultrapure water under vigorous stirring, followed by the rapid injection of 0.5 mL of 100 mM AA. Under stirring at 750 rpm, the resultant solution rapidly changed from yellow to colorless within 30 s, indicating the reduction of Au^3+^ into Au^+^. For the synthesis of L-HNS, 5 μL of L-Cys (4 mM) and 50 μL of seed solution were added to the as-obtained growth solution and stirred at 30 °C for 2 h. Interestingly, the solution gradually changed from colorless to green and realized the “L type” growth of gold horns on polyhedrons (i.e., L-HNS). Utilizing the same procedure and conditions, D-HNS and achiral HNS were synthesized by replacing L-Cys with D-Cys and DL-Cys, respectively. Meanwhile, other concentrations of L-Cys (0, 2, 6, and 8 mM) were further used to investigate the optimized condition for the best performance. Similarly, L-Cys was replaced with L-Glu and L-Phe to compare the effect of different chiral molecules on the morphology of gold nanostructures. In addition, the preparations using particle seeds and without seeds were also investigated to highlight the critical function of polyhedrons. Before characterization and application, the above gold products were centrifuged at 6000 rpm for 10 min and washed twice with distilled water, and the final precipitate was redispersed into distilled water.

### 2.3. Assessment of SERS Performance

The SERS performance of various gold nanostructures was investigated using R6G as a Raman indicator. Before that, we carefully prepared gold nanostructures-based Raman substrate on the silicon wafer via an interface self-assembly process and R6G solutions with different concentrations ranging from 10^−12^ to 10^−8^ M. Typically, 1 mL of L-HNS solution, 2 mL of dichloroethane, and 4 mL of ultrapure water were mixed in a glass vial stirring at 750 rpm for 10 s. The two-phase solution was stirred at room temperature for 1 h to form a bright film at its interface. Subsequently, 1.5 mL of n-hexane was injected along the vial wall to drive L-HNS to the interface between water and n-hexane, forming a tightly packed film of L-HNS at the interface. The resultant film was laid flat on the silicon wafer and dried in the oven at 50 °C for 2 h to obtain Raman substrates of L-HNS. To investigate SERS activity, the L-HNS substrates were incubated in the R6G solution at room temperature for 2 h to reach an equilibrium in adsorption. Then, the substrates were removed and dried at 30 °C for 6 h. SERS measurements were performed with a 50 × telephoto objective lens, and the laser excitation wavelength, power, and integration time were set at 785 nm, 2.5 mW, and 10 s, respectively. The Raman spectra were recorded at three randomly selected points, and their average value was defined as the SERS intensity for the used R6G solution at a specific concentration.

### 2.4. SERS Identification of Chiral Enantiomers

Two types of amino acid enantiomers were used as target molecules in SERS measurements to investigate the chiral response of HNSs, including L-/D-Phe and L-/D-Trp. In the experiment, the solutions containing various concentrations of enantiomers were first prepared by diluting the highly concentrated ones. Then, the Raman substrates were incubated in the enantiomer solution with a specific concentration at room temperature for 2 h. After drying at 30 °C, the enantiomer-adsorbed HNS substrates were put on the sample holder of the Raman spectrometer, and the Raman spectra were recorded as the SERS response of the enantiomer on HNS substrates. Beyond the SERS measurements for individual L-Phe and D-Phe, a mixed solution of L-Phe and D-Phe in different volume ratios (0%, 25%, 50%, 75% and 100%) was prepared, and its Raman spectra were measured on HNS substrates to further validate the discriminative ability for chiral enantiomers.

### 2.5. Preparation of Luria–Bertani (LB) Medium for E. coli

A total of 1 g of NaCl, 1 g of tryptone, and 0.5 g of yeast extract were dissolved in 100 mL of ultrapure water via sonication. The solution’s pH was adjusted to 7.0 with NaOH solution (1 M) to obtain the liquid culture medium after high-temperature sterilization at 121 °C for 20 min, which was stored in a refrigerator at 4 °C. Further, the solid medium was prepared by adding 1.5 g of agar into the liquid culture medium, which was wrapped/sterilized in an autoclave at 121 °C for 20 min and poured into sterile Petri dishes. Under strict aseptic conditions, single *E. coli* colonies on fresh striped plates were inoculated into 5 mL sterile culture medium and incubated overnight at 37 °C while continuously oscillating at 200 rpm. The growth curve was validated through periodic colony-forming unit (CFU) enumeration to ensure culture purity and reproducibility.

### 2.6. SERS Detection of E. coli

Due to the low Raman signal of bacteria, it is difficult to directly observe the SERS response of *E. coli* on HNS substrates. To this end, an additional Raman reporter molecule, 4-MPBA, was utilized to prepare 4-MPBA-modified HNSs for the SERS detection of *E. coli*. Briefly, 10 µL of 4-MPBA solution (1 mM) was added to 1 mL of HNSs solution and incubated overnight. The mixture was centrifuged to remove free 4-MPBA in the supernatant, and the precipitate was resuspended in deionized water to obtain 4-MPBA-modified HNSs. The modified HNSs were incubated with *E. coli* suspension at 30 °C for 2 h and dried at room temperature to measure SERS spectra with a Raman spectrometer. To ensure the accuracy of the results, 15 spectra were collected for each sample at different locations on the HNS substrate and then averaged for further scientific analysis.

### 2.7. Photothermal Effects of HNSs

To highlight the chiral activity of chiral HNSs in photothermal performance, left-handed circularly polarized light (LCP), right-handed circularly polarized light (RCP), and unpolarized light (UPL) were individually utilized as the light source. In the experiment, 1.5 mL of HNS solution was first sonicated in a centrifuge tube for 10 min to disperse thoroughly and then transferred to 10 mm × 10 mm quartz cuvettes. After being irradiated for 10 min by an 808 nm laser with 2.5 W/cm^2^ (LCP, RCP, or UPL), the temperature of the solutions was recorded using the hand-held thermometer. Under the irradiation of RCP, different concentrations of HNSs from 0 to 40 µg/mL were applied to investigate the effect of HNS concentration on the photothermal performance. Meanwhile, the temperature increment with time was recorded over the 10 min with a 1 min-interval. After turning off the laser, the temperature decreasing process was also observed via natural cooling at room temperature.

### 2.8. Sterilization of HNSs with/Without Light Radiation

First, the biotoxicity of HNSs was evaluated by a coincubation route. Typically, the HNSs were incubated with *E. coli* (10^4^ CFU/mL) at 37 °C for 12 h, and then the bacterial samples were applied to a solid medium using the spread plate method. The treated samples were further incubated at 37 °C for 24 h, and the number of colonies on each plate was counted using a colony counter to compare with that of the blank group. In the experiment of photothermal sterilization, HNSs were mixed with *E. coli* (10^4^ CFU/mL) and irradiated with 808 nm LCP or RCP (2.5 W/cm^2^) for 10 min, followed by cultivation at 37 °C for 12 h. Subsequently, the bacterial samples were evenly coated on the medium and incubated at 37 °C for 24 h to calculate the number of colonies on each plate to analyze the effect of circularly polarized light on sterilization efficiency.

### 2.9. Preparation of HNSs Hydrogel and Their Antibacterial Properties

To exploit the practical application of HNS’s photothermal effect, HNS hydrogel films were prepared in the presence of agarose. Briefly, a sample bottle was first preincubated at 60 °C in a water bath, and then 1 mL of agarose stock solution and 0.9 mL of diluted solution of D-HNS were added to it. The temperature was further heated to 90 °C to quicken the mixing process. After 10 min, the solution was cooled to room temperature to form a hydrogel sample containing D-HNS. Hydrogel sheets with a diameter of 2 cm were extruded using a circular mold to prepare hydrogel tablets for antimicrobial application. In a control experiment, the D-HNS solution was replaced with an equal volume of aqueous solution, and other operations were kept unchanged.

In the antibacterial experiment, a culture dish with a diameter of 2 cm for *E. coli* culture was selected, the hydrogel containing D-HNS was placed on the bandage, and the solid *E. coli* culture medium was put on the hydrogel. Then, this was illuminated with 808 nm RCP (2.5 W/cm^2^). After 1 h, the hydrogel sample was carefully removed and the number of colonies on the solid medium was recorded.

### 2.10. Characterizations

Scanning electron microscopy (SEM) images were obtained using Apreo S LoVac Field Emission Scanning Electron Microscopy manufactured by Thermo Fisher Scientific, Czech Republic. Transmission electron microscopy (TEM) images were obtained using the Talos F200X Field Transmission Electron Microscope manufactured by Thermo Fisher, USA. Ultraviolet–visible (UV–Vis) absorption spectra of the solutions containing different products were obtained using a UV1900 spectrometer manufactured by Shimadzu, Japan. Raman spectra were obtained with the Bruker Senterra II laser confocal micro-Raman spectrometer manufactured in Germany. Circular dichroism (CD) spectra were measured with the MOS-500 circular dichroism spectrometer from Biotools Inc., USA. The circularly polarized light laser (U MDL-N-808-10W) is provided by Changchun New Industrial Optoelectronic Technology Co., Ltd. in China. The size datas of the nanostructure were obtained using Zetasizer Nano ZS90 nanoparticle size and Zeta potential analyzer manufactured by Malvern, British. The temperature datas for the photothermal experiment were obtained from the handheld thermometer of Ritu Technology Co., Ltd. in Shenzhen, China.

### 2.11. g-Factor Calculation

The g-factor serves as a quantitative measure of the asymmetric degree in chiral molecules and is a critical parameter in the study of the optical properties of chiral substances. It can be calculated using the following formula:

(1) $$g-factor=2\frac{AL-AR}{AL+AR}\propto \frac{CD}{Abs}=\frac{CD\left(mdeg\right)}{32980\times Abs}$$

AL and AR are the absorption intensities of left-handed and right-handed rotation, respectively. The CD values were obtained from circular dichroism spectra, while the Abs values were measured using UV–Vis absorption spectroscopy.

To ensure data reliability, g-factor data were collected from five independent experimental replicates (n = 5), and the averaged spectrum was used as the representative spectral dataset. All spectra were processed using a smoothing to minimize noise while preserving peak features. The SD of the g-factor values was calculated through systematic error analysis, confirming the precision and reproducibility of the reported g-factor measurements.

### 2.12. Biosafety Statement

All experiments involving *Escherichia coli* in this study were conducted in accordance with the 2016 Tianjin University Laboratory Safety Management Measures. The treatment of *E. coli* is carried out under controlled laboratory conditions at Tianjin University in a certified Level II biosafety cabinet (BSC). This study did not involve human or animal pathogens.

## 3. Results and Discussion

### 3.1. Synthesis and Characterization of HNSs

In this study, Chiral HNSs were synthesized using gold polyhedrons as growth cores and Cys adsorbed on their surfaces to induce the continuous deposition of Au atoms. The formation of gold polyhedrons initiated from Au_25_ nanoclusters, accompanied by a distinct color transition of the solution from yellow to red, is indicative of nanoparticle growth (Figure S1a,b). In this system, the synergistic interplay of CTAB as a stabilizing surfactant, HAuCl_4_ as the gold precursor, and AA as the reducing agent establishes the foundational reaction environment. Notably, Cys is a well-established chiral amino acid ligand that has been widely employed as a functional ligand in the biological functionalization of nanomaterials due to its antioxidant properties [28,29]. In chiral gold nanoparticle synthesis, the thiol group of Cys forms Au–S bonds, which direct the asymmetric deposition of gold atoms via strong ligand binding, driving anisotropic growth of protruding angles on gold polyhedrons and thereby determining the morphological complexity of chiral nanostructures.

To achieve precise morphological control of chiral HNSs, we systematically engineered the chemical environment for gold nanoparticle synthesis using an improved seed-mediated growth methodology. The design of the growth solution was based on established strategies for chiral gold nanomaterials [14]. Specifically, the volumes of CTAB (100 mM) and HAuCl_4_ in the growth medium were set to 0.8 mL and 0.2 mL, respectively. This parameter was determined through the literature review and preliminary experimental validation, as a high CTAB concentration (≥100 mM) preferentially adsorbs on {110} crystal facets, effectively suppressing non-directional nucleation and heterogeneous growth to provide a stable template for multi-branched nanostructure formation. The low concentration of HAuCl_4_ (10 mM) was deliberately selected to balance gold ion supply with structural stability, avoiding excessive precursor-induced overgrowth or destabilization of the branched architecture. Moreover, the synergistic interplay between CTAB and AA was utilized to modulate the growth kinetics [30]. By carefully controlling the AA concentration (100 mM), we maintained a slower reduction rate, thereby highlighting the regulatory roles of CTAB and the chiral ligand Cys in morphology evolution. Experimental observations indicate that Cys, functioning as a chiral inducer, serves as the critical parameter for tuning the chiral activity and symmetry of the final product. Thus, the fixed ratio of CTAB to HAuCl_4_ establishes a stable structural foundation for this regulatory process, whereas the Cys concentration emerges as the primary variable for optimizing chiral activity and morphology.

**Figure 1:**
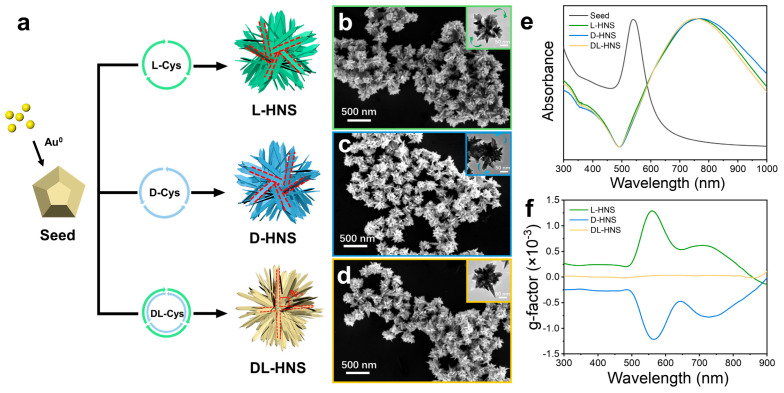
Growth of gold horns on the surface of gold polyhedrons and their morphological characteristics. (**a**) Schematic representation for growing gold horns on polyhedron in the presence of L-cysteine (L-Cys), D-cysteine (D-Cys), and DL-cysteine (DL-Cys), which produce chiral and achiral horned gold nanostructures (HNS). (**b**–**d**) SEM images of L-HNS, D-HNS, and DL-HNS, which are synthesized using 4 mM L-Cys (**b**), 4 mM D-Cys (**c**), and 4 mM D-Cys (**d**), respectively. Inserts: the corresponding TEM images of different HNSs. (**e**) Normalized UV–Vis absorption spectra of gold polyhedron seeds, chiral and achiral HNSs. (**f**) g-factor spectra of L-HNS, D-HNS, and DL-HNS, indicating that L-HNS and D-HNS exhibit larger and opposite chirality while DL-HNS have no chirality. The g-factors of L-HNS at 560 nm (mean = 0.0013, SD = 0.00000387, n = 5). The g-factors of D-HNS at 560 nm (mean = −0.0013, SD = 0.00000312, n = 5).

As illustrated in Figure 1a, the chirality of Cys dictates the directional growth of horns on gold polyhedron seeds through stereoselective interactions. Specifically, L-Cys drove a right-handed helical deposition of Au atoms (Figure 1b): the SEM image shows homogeneous L-Cys-induced horned nanostructures (L-HNS), while the high-resolution TEM image of individual particles highlights clockwise-arranged protruding horns (Figure 1b, inset). Conversely, D-Cys induced a mirror-image left-handed growth pattern, with the TEM image showing counterclockwise horn arrangements (Figure 1c). In contrast, DL-Cys eliminated chirality through racemic competition, resulting in symmetrically aligned horns extending from the polyhedral core (Figure 1d). Despite their distinct geometric chirality, all three nanostructures exhibit identical LSPR peaks at ~780 nm (Figure 1e), confirming structural similarity in overall dimensions. Interestingly, the critical differentiation emerges in their chiroptical responses: L-HNS and D-HNS displayed perfect mirror-symmetric anisotropy factor (g-factor) spectra with opposite peaks at ±0.0013 (λ ≈ 560 nm), directly mirroring their right- and left-handed rotational horn architectures. DL-HNS, lacking chiral asymmetry, showed no detectable g-factor signal (Figure 1f). This structural optical correlation determines that the rotating arrangement of horns programmed by L-/D-Cys during growth is linked to their chiral origin. Conversely, DL-Cys disrupts this directional control through competitive binding. In addition, Figures S2 and S3 demonstrate that the synthesized HNS exhibits highly reproducible chiral activity and morphological features, confirming the reliability of the synthesis scheme. Their chiral activity and morphology reach a stable state within 120 min.

During the process of horns growth, the morphology of gold seeds and the type of chiral molecules profoundly impact the morphology and chiral activity of HNSs. As shown in Figure S4a, the green solution of L-HNS was obtained in the presence of gold polyhedrons and L-Cys and exhibited excellent chiral optical activity. If the seeds were replaced with gold nanospheres, the solution’s product color would have shown a slighter green, but the extreme value of the g-factor would have significantly decreased from 0.0013 to 0.0004 (Figure S4b). Furthermore, the blank sample without adding seeds only produced large precipitates with no g-factor spectral peaks. The above comparisons demonstrate the critical role of seeds in the synthesis system: the seeds with multiple facets are one source of enhanced chiral activity (Figure S4c). In addition, the type of chiral ligand also affects the structural changes in the product. Typically, L-Cys can synthesize uniform horned gold nanostructures (Figure S5). Additionally, L-Cys was replaced by L-glutamic acid (L-Glu) during the synthesis process, resulting in the growth of irregular-shaped particles, and no chiral signal was observed (Figure S6a,b). Spike-like protrusions were obtained to exhibit no chiral activity if using L-Pen as a chiral molecule (Figure S7a,b). This indicated that thiol groups played a central role in the growth and formation of HNSs. The amino acids without thiol groups, such as Glu, will not result in horn coverage structures. Although Pen contains thiol groups, its rigid molecular structure causes difficulty in adsorbing in an orderly manner on the surface of gold seeds. Thus, the product cannot exhibit chiral activity. Therefore, chiral Cys and polyhedron seeds have contributory effects in the formation of chiral HNSs.

**Figure 2:**
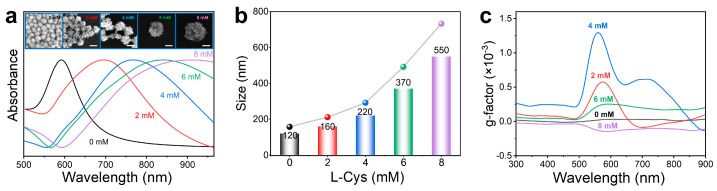
Effect of L-Cys concentrations on size and chirality of as-synthesized HNSs. (**a**) Normalized UV–Vis absorption spectra of HNSs synthesized using 0, 2, 4, 6, and 8 mM L-Cys. Insets: SEM images of the as-obtained HNSs using different concentrations of L-Cys from 0 to 8 mM (scale bar: 200 nm). (**b**) Evolution of the average size of HNSs measured by a nanoparticle size analyzer with an increment of L-Cys concentrations. (**c**) g-factor spectra of HNSs synthesized in the presence of different concentrations of L-Cys. It can be seen that the gradual increment of L-Cys continuously increases the size of HNSs. Still, their chirality exhibits an initial increment and subsequent decreasing trend (i.e., 4 mM L-Cys induces the largest chirality in HNSs).

Furthermore, the effect of the concentration of Cys on the morphology and chiral activity of HNS was explored. As shown in Figure 2a, within the range of 0–8 mM, the absorption peak underwent a continuous red shift with increasing L-Cys concentration. The SEM images show the change in morphological features: when the concentration of L-Cys was 0 mM, irregularly shaped nanoparticles without distinct horns were formed. As the L-Cys concentration increased to 2 mM, a limited number of coarse horns emerged on the nanoparticle surfaces, indicating that L-Cys began to mediate the site-specific deposition of Au atoms on the gold polyhedrons surfaces. This morphological evolution reached optimal expression at 5 mM L-Cys concentration, where nanoparticles exhibited uniform horn coverage with well-defined horn structures, demonstrating the critical role of L-Cys concentration in directing precise morphological control during nanoparticle growth. However, when the L-Cys concentration was further increased to 6 mM and 8 mM, the horns transitioned toward spiky shapes, accompanied by a notable increase in surface coverage. During the overall rise in L-Cys concentration, the product’s particle size also grew from 120 nm to 550 nm, corresponding to the red shift in the absorption peak (Figure 2b).

The spectral behavior of the g-factor further confirms that the chiral activity of L-HNS originates from its inherent structural chirality rather than surface-adsorbed chiral ligands. As shown in Figure 2c, the g-factor exhibits a non-monotonic dependence on L-Cys concentration (0–8 mM), reaching a maximum value of ± 0.00130 at 560 nm under 4 mM L-Cys before declining at higher concentrations. Most importantly, despite the increased ligand loading, the observed decrease in g-factor amplitude at 6 mM and 8 mM L-Cys excludes molecular adsorption as a chiral source. Based on the experimental results, it can be concluded that the chiral optical properties of chiral HNSs originate from their inherent chiral architecture, the formation of which is attributed to the enantiomer-induced asymmetric deposition of gold atoms by L-/D-Cys enantiomers. Mechanistically, the thiol groups of Cys form Au–S bonds with high-energy facets, selectively passivating these regions to inhibit Au deposition. Concurrently, steric hindrance from the amino/carboxyl groups and the molecular flexibility of Cys induce facet-dependent adsorption asymmetry. This asymmetric passivation directs newly reduced Au atoms to deposit along enantiomer-specific helical trajectories (clockwise for L-Cys and counterclockwise for D-Cys), resulting in the growth of geometrically chiral horned architectures. Optimal chiral activity at 4 mM L-Cys reflects balanced ligand coverage that maximizes facet selectivity while maintaining growth anisotropy. However, ligand saturation homogenizes adsorption across all crystallographic facets at elevated concentrations (6 mM and 8 mM), eliminating facet selectivity. This results in isotropic Au deposition, which disrupts helical symmetry and diminishes structural chirality, as evidenced by attenuated g-factor signals.

### 3.2. SERS Performance of Chiral HNSs

Chiral nanostructures critically enable enantioselective sensing, biomedicine, and asymmetric catalysis through their asymmetric geometries that drive molecule-specific interactions. Chiral HNSs developed in this article exhibited excellent SERS performance. The horn structure of chiral HNSs provides more surface areas and hotspots for Raman enhancement and exhibits strong localized surface plasmon resonance effects in the visible and near-infrared bands. It matches the resonance energy with a Raman laser (785 nm) to amplify excited and scattered photons [31,32,33].

Using three-phase interfacial self-assembly, HNS self-assembles into dense membranes at the n-hexane/water interface, driven by interfacial tension gradients between n-hexane/water and dichloroethane/water. The gold film is deposited horizontally onto the silicon wafer surface to fabricate the SERS-active substrate. To screen SERS substrates with strong signals and high chiral activity, L-HNS synthesized at varying L-Cys concentrations (0–8 mM) were evaluated using 10^−10^ R6G. As shown in Figure S8a, L-HNS synthesized with 6 mM L-Cys exhibited the most substantial SERS enhancement. In addition, L-HNS synthesized with 4 mM L-Cys was also significantly enhanced due to its dense angular protrusions, which maximized molecular adsorption and achieved optimal LSPR matching at the 785 nm excitation wavelength. However, at 8 mM L-Cys, the SERS performance was degraded due to the loss of plasmonic coupling caused by the mismatch of LSPR and Raman excitation wavelengths and excessive nanoparticle aggregation. After that, L-, D-, and DL-HNS synthesized with 4 mM Cys were individually used as Raman substrates to test a 10^−10^ M R6G solution, and it was found that the SERS effects of the three substrates were almost the same (Figure S8b). Chiral HNSs Raman substrates have the same Raman enhancement effect for achiral molecules. Given our focus is on chiral active HNSs, we have chosen 4 mM Cys synthesized HNSs with the best chiral activity (g-factor at 560 nm wavelength: ±0.0013) for further research.

**Figure 3:**
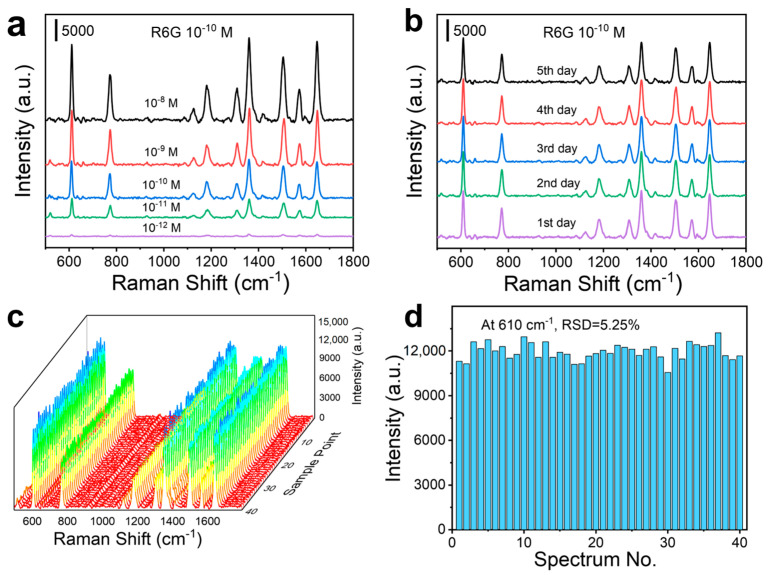
SERS measurements of R6G on L-HNS substrates (4 mM L-Cys is used for synthesizing the L-HNS). (**a**) SERS spectra for different concentrations of R6G on L-HNS Raman substrate after adsorption for 2 h. (**b**) SERS spectra of 10^−10^ M R6G on L-HNS substrate with an increment of adsorption time. (**c**) SERS spectra of 10^−10^ M R6G on L-HNS substrate at 40 randomly selected spots. (**d**) Comparison of the Raman intensity at 610 cm^−1^ for 40 SERS spectra, which generalizes a relative standard deviation (RSD) of 5.25%.

To evaluate the sensitivity, stability, and reproducibility of HNS SERS substrates, the Raman analysis of 10^−10^ M R6G was conducted by employing L-HNS synthesized with 4 mM L-Cys. Adsorption saturation was achieved within 2 h (peak variation < 5% at 3 h; Figure S9a). In the experimental design, R6G was tested over a concentration gradient from 10^−12^ M to 10^−8^ M using the L-HNS substrates with 2 h adsorption (Figure 3a). As shown in Figure S9b, quantitative analysis revealed a linear relationship between the SERS intensity at 610 cm^−1^ and the logarithmic R6G concentration (10^−12^–10^−8^ M), yielding the equation y = 4976.955x + 59,983.270 with R^2^ = 0.997, demonstrating the ability of quantitative analysis. The substrate reliably detected 10^−12^ M R6G with a signal-to-noise ratio of (SNR) 5.1 (Figure S10a). However, no discernible Raman signal was observed at 10^−13^ M (Figure S10b), establishing 10^−12^ M as the detection sensitivity (SNR should be greater than 3).

Furthermore, the Raman enhancement factor (EF) of L-HNS at an analyte concentration of 10^−12^ M was calculated to be 2.8 × 10^8^ using the characteristic R6G peak at 610 cm^−1^. The calculation formula is shown below:

(2) $$EF=\left(\frac{I_{\mathrm{SERS}}}{N_{\mathrm{SERS}}}\right)/\left(\frac{I_{\mathrm{bulk}}}{N_{\mathrm{bulk}}}\right)$$

The detailed parameter descriptions and calculation procedures are provided in Note S1 and Table S1 of the Supplementary Information.

For the stability test of L-HNS substrate, the L-HNS substrate stored at room temperature for 0–6 days showed a change of less than 5% in SERS signal intensity for R6G at 10^−10^ M (Figure 3b). In addition, reproducibility assessment across 40 random measurement points (Figure 3c) confirmed substrate uniformity, with a relative standard deviation (RSD) of 5.25% for the 610 cm^−1^ peak intensity (Figure 3d).

### 3.3. Recognition of Amino Acid Enantiomers by Chiral HNSs

Chiral HNSs demonstrate remarkable SERS performance and chiral activity, which are critical for enantiomeric amino acid recognition. Notably, the spiky architecture of the HNSs synthesized in this work presents distinct advantages over conventional chiral gold nano systems. The three-dimensionally branched structure enhances the electromagnetic field via LSPR hotspots, increases the active surface area for molecular adsorption, and facilitates orientation-dependent chiral discrimination due to the anisotropic distribution of functional ligands. These structural characteristics, compared to helical chiral gold nanoparticles reported in the literature, collectively improve the sensitivity and selectivity of enantiomeric amino acid detection. It should be noted that the Raman spectra of pure solid powders of D-Phe and L-Phe are the same (Figure S11), but there is a difference in the intensity of Raman scattering signals when their solutions are adsorbed onto chiral HNSs. All Raman intensity comparisons utilize the Raman peak intensity of Phe at 1001 cm^−1^ as the reference standard. As shown in Figure S12, the product synthesized without L-Cys (0 mM) exhibited the weakest SERS performance and no chiral discrimination capability for Phe enantiomers. While 2 mM L-Cys synthesized L-HNS showed a 1.6-fold SERS intensity difference between L-Phe and D-Phe, its overall enhancement remained suboptimal. Meanwhile, 6 mM and 8 mM L-Cys synthesized L-HNS displayed more vigorous SERS activity but lost enantiomeric discrimination ability.

**Figure 4:**
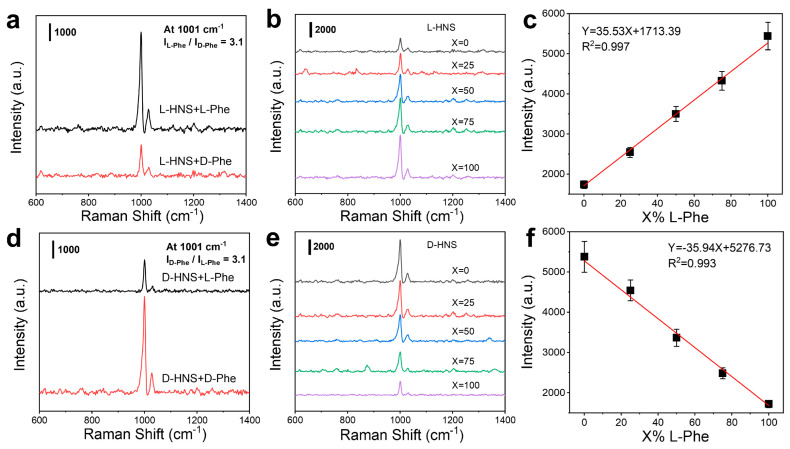
Diverse sensitivity of chiral HNSs for enantiomers. (**a**) SERS spectra of 0.5 mM L-Phe and 0.5 mM D-Phe on L-HNS substrate. (**b**) SERS spectra of a mixed solution of L-Phe and D-Phe on L-HNS Raman substrate, in which X% is the content of L-Phe in the mixed solution (the total concentration of L-Phe and D-Phe is 0.5 mM). (**c**) Linear relationship between Raman intensity at 1001 cm^−1^ and L-Phe content (mean ± SD, n = 5). (**d**) SERS spectra of 0.5 mM L-Phe and 0.5 mM D-Phe on D-HNS substrate synthesized under the induction of 4 mM D-Cys. (**e**) SERS spectra of mixed L-Phe and D-Phe on D-HNS substrate, in which X% is still the content of L-Phe in the mixed solution. (**f**) Linear relationship between Raman intensity at 1001 cm^−1^ and L-Phe content (mean ± SD, n = 5). In all measurements, the adsorption time is 2 h.

It is worth noting that the L-HNS synthesized with 4 mM L-Cys exhibited the best chiral recognition ability, with the SERS intensity of L-Phe being 3.1 times higher than that of D-Phe (Figure 4a). In contrast, D-HNS demonstrated that D-Phe adsorption resulted in a 3.1-fold stronger Raman signal compared to L-Phe (Figure 4d), a difference that was statistically significant (Figure S13, *** *p* < 0.001), which confirms the reliability of the data. This experimental result suggests that the chiral atomic arrangement within inorganic crystals (L-/D-HNS) may induce asymmetric adsorption of chiral molecules, and the chirality of substrates and analytes could lead to inhomogeneous adsorption patterns or orientation-dependent electromagnetic field enhancement, thus generating differences in Raman signal intensities [34,35]. Moreover, this chiral recognition capability directly correlates with measured g-factor magnitudes, where the enhanced chiral response determines excellent enantioselectivity. To quantify recognition performance, we analyzed mixed solutions of L-Phe and D-Phe (total concentration: 0.5 mM) with varying L-Phe volume fractions X% (X = 0, 25, 50, 75, and 100). Following 2 h adsorption on L-HNS and D-HNS substrates, the SERS intensity of the L-HNS substrate at 1001 cm^−1^ increased with an increase in X%, while the opposite was true on D-HNS (Figure 4b,e). In addition, the linear fitting relationship between I*_1001_* (signal intensity at 1001 cm^−1^) and X was plotted separately (Figure 4c,f). This confirms that chiral HNSs exhibit excellent enantioselectivity and quantitative discrimination ability for pure chiral amino acids.

The chiral recognition versatility of HNSs was further validated using additional enantiomers of tryptophan (Trp) (Figure S14). Collectively, L-/D-HNS substrates leverage selective intermolecular interactions to achieve precise discrimination of amino acid enantiomers through enantiomer-dependent Raman signal modulation, thus establishing a general platform for chirality-based molecular sensing.

### 3.4. Discrimination of Bacteria by Chiral HNSs

The enantioselective recognition capability of chiral HNSs toward amino acids suggests promising applications in bacterial detection. Bacterial cell walls contain abundant chiral components, including peptidoglycan and amino acids [36,37]. Leveraging the selective recognition capability of chiral HNSs substrates, these chiral motifs can be specifically captured and detected through SERS with enhanced sensitivity.

**Figure 5:**
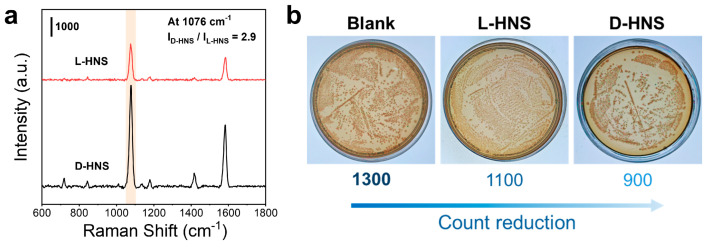
Adsorption affinity of chiral HNSs to bacteria *E. coli* and their biotoxicity. (**a**) SERS spectra of *E. coli* on L-HNS and D-HNS, in which the incubation time is 2 h, and a Raman probe 4-MPBA is used. (**b**) The average colony count of *E. coli* was measured in three experimental groups (n = 5): *E. coli* incubated with L-HNS, *E. coli* incubated with D-HNS, and a blank group without chiral HNSs. As seen, D-HNS has a much higher affinity to *E. coli*. than L-HNS, and they have a slight bactericidal ability via interaction.

In this section, 4-MPBA was introduced as a “catcher” to enforce the binding force between chiral HNSs and bacteria, and meanwhile as a Raman tag to provide SERS signal for amplifying the differences among the bacterial “fingerprints”. The 4-MPBA molecule contains three functional groups: the thiol group that forms Au–S bonds with gold, enabling surface modification of gold nanoparticles; the boric acid groups that bind with peptidoglycans in bacterial cell walls to form cyclic boronic acid esters, thereby achieving bacterial identification; and the benzene ring, which significantly amplifies the SERS signal captured by bacteria [38]. For *E. coli* detection, chiral 4-MPBA-modified HNSs were fabricated as SERS substrates, immersed in bacterial suspension for 2 h adsorption, and analyzed after drying at room temperature. As shown in Figure S15a, unlabeled D-HNS substrates incubated with *E. coli* culture medium exhibit no detectable SERS peaks (red curve), demonstrating that non-functionalized D-HNS cannot directly identify bacterial signatures. In contrast, the D-HNS substrate modified with 4-MPBA exhibited a distinct *E. coli* SERS “fingerprint” peak (black curve), significantly enhancing its binding affinity with bacteria. However, when 4-MPBA was incubated with *E. coli* alone for Raman testing, no characteristic signals were generated (blue curve), demonstrating the importance of the D-HNS substrate. To further identify the distinctive peaks of *E. coli*, we first tested the SERS signal of 4-MPBA-modified D-HNS, which produced only the characteristic peaks located at 1078 cm^−1^, 1179 cm^−1^, and 1538 cm^−1^. In contrast, 4-MPBA-modified D-HNS incubated with *E. coli* exhibits distinct bacterial “fingerprint” peaks at 718 cm^−1^, 845 cm^−1^, and 1417 cm^−1^ (Figure S15b). These vibration modes correlate with key bacterial wall components and excreted metabolites, establishing characteristic spectral signatures for rapid bacterial identification (Table S2) [36,37,39]. Subsequently, we detected *E. coli* using L-HNS and D-HNS substrates modified with 4-MPBA, respectively. Given the enhancing effect of 4-MPBA on the Raman signal of *E. coli*, all Raman intensity comparisons were mainly based on the peak at 1076 cm^−1^ of 4-MPBA. As shown in Figure 5a, the SERS signal of the D-HNS substrate (modified with 4-MPBA) is 2.9 times higher than that of the L-HNS counterpart, demonstrating its stronger affinity for bacterial components. A t-test was further conducted to confirm the statistical significance of this difference, yielding a p-value of *** *p* < 0.001 and confirming the data’s reliability (Figure S16). The observed disparity is likely attributed to the enhanced stereoselective interaction between D-HNS and D-amino acids within the bacterial peptidoglycan layer [40].

Based on the above experimental results, chiral HNSs exhibit a significant affinity for *E. coli*. We attempted to introduce HNSs into the application of sterilization. To evaluate the antimicrobial capacity of HNSs against *E. coli*, we co-incubated L-HNS and D-HNS with bacterial suspensions for 12 h alongside a blank control. The mixtures were then plated on solid medium and cultured at 37 °C for 24 h. As shown in Figure 5b, the blank group exhibited ~1300 colonies, while L-HNS and D-HNS treatments reduced colony counts, with D-HNS demonstrating the most potent antibacterial activity. 9 further illustrates the significant differences in colony counts among the three experimental groups using statistical methods (***p < 0.001). This effect likely arises from D-HNS’s dual mechanism of bacterial affinity-driven adsorption and physical puncture. Based on this enhanced targeting capability, D-HNS was prioritized for subsequent antimicrobial investigations. However, the observed sterilization efficiency remained suboptimal, indicating requirements for further refinement.

### 3.5. Dual Antimicrobial Activity of Chiral HNSs

Effective management of bacterial infections requires precise antimicrobial interventions to inhibit pathogen proliferation and dissemination. Nanomaterial-mediated photothermal therapy (PTT) has excellent potential for antimicrobial applications [41,42]. Gold nanomaterials have emerged as leading candidates for photothermal antibacterial research due to their outstanding LSPR properties and inherent biocompatibility [22,43]. Chiral HNSs have established a synergistic platform for the physical destruction of chiral gold nanoparticles and CPL-mediated photothermal sterilization through their near-infrared LSPR response and optimized photothermal conversion efficiency. Notably, the spiky architectures of chiral HNSs offer distinct advantages over conventional chiral gold nanomaterials. The three-dimensionally branched morphology enhances light absorption and local hotspot distribution, while the sharp tips generate stronger plasmonic heating due to field concentration. Additionally, the hierarchical nanostructures facilitate deeper thermal penetration into bacterial biofilms. These structural features, combined with the polarization-dependent photothermal response, enable chiral HNSs to synergistically improve photothermal sterilization efficiency compared to traditional sterilization materials, while maintaining enantioselectivity in bacterial targeting.

Integrating CPL into photothermal sterilization represents a promising strategy to achieve enhanced bactericidal efficacy and biosafety. However, left-handed circularly polarized light (LCP) and unpolarized light (UPL) require high-power irradiation to achieve equivalent bactericidal effects due to poor stereoselective resonance with D-amino acid-rich bacterial cell walls, risking non-targeted thermal damage to surrounding tissues. In contrast, right-handed circularly polarized light (RCP) selectively targets bacterial membranes through chiral-matched interactions with D-amino acids in peptidoglycan layers. Therefore, RCP is an ideal choice for energy delivery and biocompatibility.

**Figure 6:**
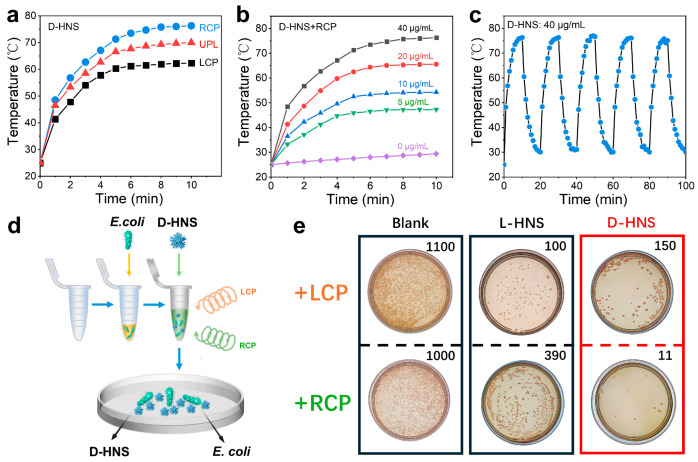
Photothermal capability of D-HNS and bactericidal ability under irradiation of polarized light. (**a**) Temperature-rising curves of D-HNS solution with increasing time under RCP, LCP, and UPL laser irradiation (2.5 W/cm^2^). (**b**) Temperature-rising curves of solution with different D-HNS concentrations under RCP irradiation. (**c**) Temperature change of 40 μg/mL D-HNS solution under RCP irradiation for 5 switching cycles. (**d**) Schematic of photothermal sterilization on D-HNS by using circularly polarized light. (**e**) Following a 10 min incubation of *E. coli* with chiral HNSs under CPL irradiation, the bacterial suspension was inoculated onto solid culture medium and subsequently cultured at 37 °C for 24 h. As revealed, the combination of D-HNS and RCP exhibits the best performance in sterilization. The colony count is based on the average value (n = 5).

Significant polarization-dependent photothermal responses were observed between D- and L-HNS under CPL irradiation. Under RCP irradiation, D-HNS solutions exhibited markedly accelerated heating rates compared to UPL and LCP, with LCP showing the slowest temperature rise (Figure 6a). Conversely, L-HNS displayed an inverse polarization-dependent response, achieving optimal photothermal efficiency under LCP (Figure S18a). DL-HNS showed identical thermal responses under both CPL and UPL conditions (Figure S18b), demonstrating polarization-independent behavior due to its achiral structural symmetry. Given the biocompatibility of RCP and D-HNS’s enhanced binding affinity for *E. coli*, these components were prioritized for our photothermal conversion tests. As shown in Figure 6b, the photothermal equilibrium temperature continuously increases with the concentration of D-HNS from 0 to 40 μg/mL. Moreover, we conducted five on/off cycles of RCP irradiation on the D-HNS solution to determine its excellent photothermal repeatability and stability (Figure 6c).

Subsequently, to calculate the photothermal conversion efficiency (η) of D-HNS under RCP irradiation, we determined the heat transfer time constant (τs) as 250.347 s through linear regression analysis of the cooling curve (Figure S19). Then we calculated the photothermal conversion efficiency using the following derived formula:

(3) $$\eta =\frac{\left[hS\left(T_{max}-T_{\mathrm{surr}}\right)-Q_{dis}\right]}{I\left(1-10^{-A808}\right)}$$

The parameter definitions of the formula are detailed in Note S2, and the photothermal conversion efficiency of D-HNS under RCP irradiation was calculated to reach 51.85%.

To assess the photothermal antibacterial performance of CPL in combination with chiral HNSs, *E. coli* suspensions were first combined with chiral HNSs, and the mixture was then irradiated with CPL (λ = 808 nm, 2 W/cm^2^) for 10 min and mixed for 12 h. Afterwards, it was coated on a solid culture medium and incubated at 37 °C for 24 h (Figure 6d). Figure 6e shows the colony count of the blank group and the addition of chiral HNSs, and the *E. coli* bacterial solution incubated with chiral L-/D-HNS exhibited a significant antibacterial response after CPL irradiation. A quantitative analysis of bacterial colonies showed that the survival rate in a chiral HNSs medium was significantly reduced compared to that of the blank group. Under LCP irradiation, the L-HNS-treated group exhibited the lowest colony count (~100), achieving a sterilization efficiency of 92% compared to the no-treatment baseline (no HNS, no light: ~1300 colonies). However, D-HNS exhibited the most substantial sterilization effect under RCP irradiation in all the controls. The sterilization rate reached 99%. The one-way ANOVA in Figure S20 shows significant differences in the data (*** *p* < 0.001), thereby confirming the reliability of the experimental results. Combining the results of the experiments in the previous section, the enhanced sterilization efficacy of D-HNS stems from its specific adsorption to bacterial D-amino acid residues, which strengthens membrane anchoring and facilitates localized thermal disruption of cell walls through photothermal conversion. The experimental results indicate that the synergistic effect of RCP and D-HNS can stimulate the photothermal sterilization mechanism. In contrast, the sterilization effect is limited due to the mismatch of LCP rotation direction. Therefore, combining RCP and D-HNS is ideal for optimizing antibacterial strategies.

### 3.6. Antimicrobial Properties of D-HNS-Doped Hydrogel Film

**Figure 7:**
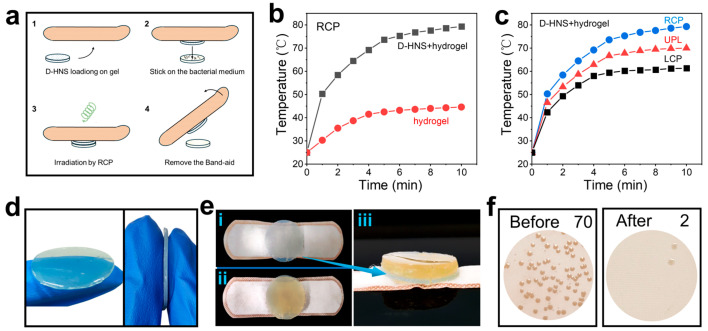
The practical application expansion of photothermal sterilization. (**a**) Schematic diagram of photothermal sterilization of D-HNS-doped hydrogel film under RCP irradiation. (**b**) Temperature rise curve of D-HNS-doped hydrogel film and blank hydrogel under RCP (2.5 W/cm^2^) irradiation. (**c**) Temperature rise curve of D-HNS-doped hydrogel film under RCP, LCP, and UPL (2.5 W/cm^2^). (**d**) Pictures of D-HNS-doped hydrogel film. (**e**) Pictures of D-HNS-doped hydrogel film and *E. coli* solid medium, (**e**-**i**) Separate top view of D-HNS-doped hydrogel film, (**e**-**ii**) top view of D-HNS-doped hydrogel film fitting with *E. coli* solid medium, and (**e**-**iii**) side view of D-HNS-doped hydrogel film fitting with *E. coli* solid medium. (**f**) The average colony count on solid culture medium before and after RCP irradiation (n = 5).

Hydrogel is a biomaterial capable of cell culture, tissue engineering, and drug delivery [44]. To expand the application scope of chiral HNSs materials, this study successfully developed an agarose-based composite hydrogel carrier incorporating chiral HNSs, constructing a directionally responsive antibacterial film through precise manipulation of photothermal conversion characteristics. As illustrated in Figure 7a, the experimental procedure comprised fabricating a D-HNS-doped hydrogel film, integrating it into a medical-grade bandage, and placing the solid *E. coli* culture medium on the hydrogel film. After 1 h of irradiation with RCP, the hydrogel sample was carefully removed and the number of colonies on the solid medium was recorded.

The photothermal performance test showed that the D-HNS-doped hydrogel film produced a significantly enhanced thermal effect under the irradiation of RCP, and its steady-state temperature was 77.8% higher than that of the blank hydrogel film (Figure 7b). Meanwhile, the D-HNS-doped hydrogel film had a polarization-dependent response (Figure 7c), which was consistent with the photothermal characteristics of the previous L-/D-HNS nanomaterials and confirmed that the chiral matching RCP irradiation could activate the surface plasmon resonance effect of the material, providing a basis for accurate photothermal treatment.

Following photothermal conversion testing, sterilization studies of the prepared hydrogel film were conducted. As depicted in Figure 7d, the hydrogel displays a light green disk-shaped morphology (2 cm diameter, 1 mm thickness) with a three-dimensional network structure that effectively immobilizes HNS nanoparticles. Figure 7e depicts the integration of the hydrogel with *E. coli* culture medium. The yellow solid culture medium (bacterial density ~70 colonies) is presented in the left panel of Figure 7f. Subsequently, the hydrogel-culture medium composite was irradiated with RCP for 1 h, after which the hydrogel film was carefully removed. The results revealed a reduction in bacterial colonies to two, showing that the sterilization rate reaches 97% (Figure 7f, right), demonstrating the D-HNS-doped hydrogel film’s excellent antibacterial effect under photothermal-activated RCP treatment. This finding highlights the practical application potential of chiral HNSs in photothermal sterilization.

## 4. Conclusions

This study developed chiral horned gold nanostructures (HNS) via a seed-mediated secondary growth method. The geometric chirality originating from horn-rotational arrangement directly governs the optical chiral signals. This three-dimensional architecture provides abundant SERS-active sites, enabling efficient bacterial detection and amino acid enantiomer discrimination. Specifically, D-HNS displays enhanced affinity toward *E. coli*, achieving fingerprint-level SERS identification of bacterial components. Furthermore, the chiral HNSs exhibit polarization-dependent photothermal conversion under CPL. By combining targeted bacterial adsorption with RCP irradiation, D-HNS achieves synergistic sterilization through physical structural disruption and localized photothermal effects. The integration of D-HNS into agarose hydrogel matrices further demonstrates scalable applications for biomedical sterilization. Future research will focus on elucidating the chiral molecule recognition mechanism in gold nanomaterials, further optimizing photothermal stability, and advancing the applications of chiral recognition and antibacterial therapy.

## Data Availability

The original contributions presented in this study are included in the article/Supplementary Materials. Further inquiries can be directed to the corresponding author.

## Supplementary Materials

The following supporting information can be downloaded at: https://www.mdpi.com/article/10.3390/ma18112627/s1.
