# Supplementary material for: Chiral Growth of Gold Horns on Polyhedrons for SERS Identification of Enantiomers and Polarized Light-Induced Photothermal Sterilization
Source: Materials (Basel). 2025 Jun 4;18(11):2627. doi: 10.3390/ma18112627 (PMC12155605; doi:10.3390/ma18112627)
Supplement: Supplementary file 1 [file materials-18-02627-s001.zip › materials-3643078-supplementary.pdf]

Supplementary Materials

Chiral Growth of Gold Horns on Polyhedrons for SERS Identification of Enantiomers and Polarized Light-Induced Photothermal Sterilization

Bowen Shang and Guijian Guan *

Institute of Molecular Plus, Tianjin University, Tianjin 300072, China
* Correspondence: guijianguan@tju.edu.cn

Statistical Analysis

Raman spectra were pre-processed with multipoint linear baseline correction using HORIBA LabSpec software to correct for background noise. The spectral data of g-factor and extinction were processed using Microsoft Excel and OriginPro software.

Statistical analysis was performed using GraphPad Prism 9 version 9.0.2 (134) for MacOs. All spectral data in the manuscript, including g-factor spectra, UV-Vis absorption spectra, and Raman spectra, were obtained from five independent replicate measurements, with the mean value used as the final data. Error bars represent the standard deviation (SD) calculated from at least five average spectra, and the data are presented as the mean ± SD. Statistical significance of the SERS signal intensity was assessed using an independent t-test during comparisons. $p < 0.05$ was considered statistically significant.

All bacterial colony counts (colony number per plate) were determined in five measurements and expressed as mean ± SD. Statistical significance between groups was evaluated using one-way ANOVA, multiple comparison correction and the Bonferroni post-hoc test, with $p < 0.05$ considered statistically significant.

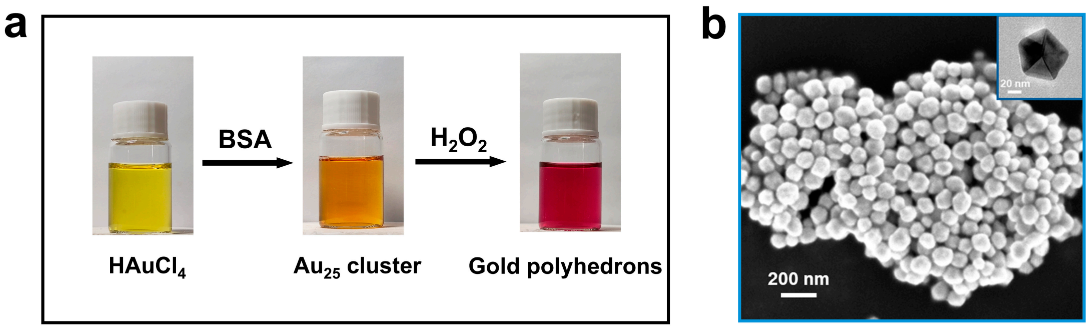

Figure S1. (a) Optical demonstration from $HAuCl_4$ to synthesize Au_{25} clusters and gold polyhedrons. (b) SEM and TEM (inset) images of gold polyhedrons for presenting their five-fold symmetric structure.

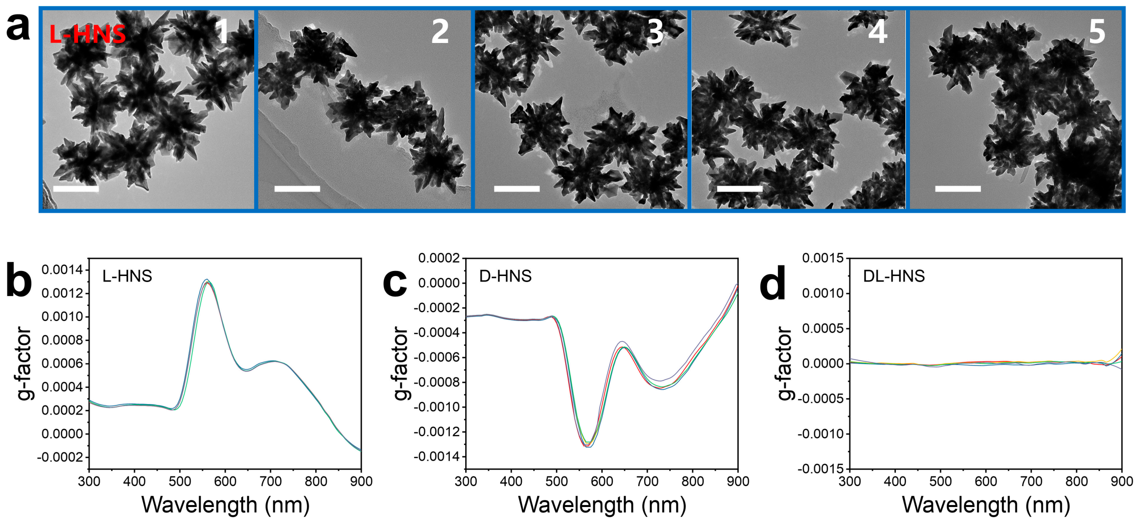

Figure S2. **(a)** TEM images of L-HNS morphology obtained from five independent experiments reveal excellent size uniformity and consistent morphological features. The g-factor spectral profiles of products from five independent syntheses of (b) L-HNS, (c) D-HNS, and (d) DL-HNS exhibit a high degree of spectral overlap, confirming experimental reproducibility.

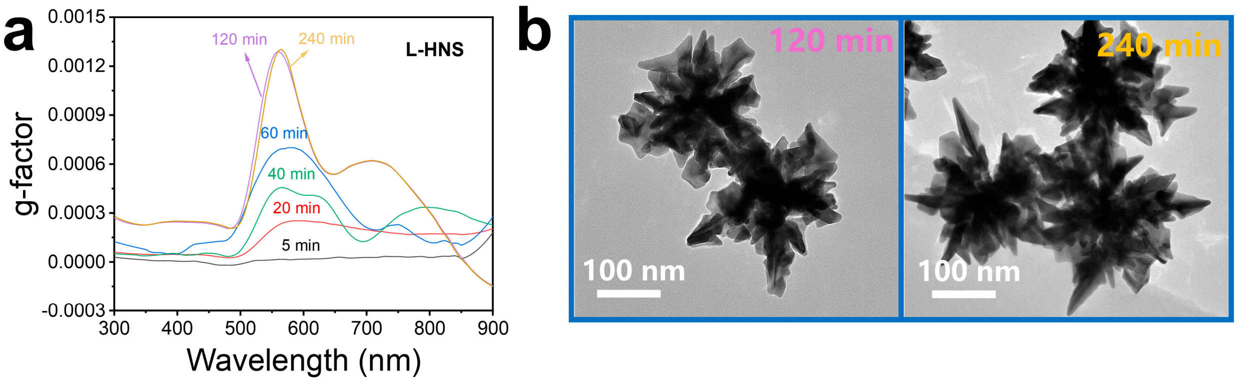

Figure S3. **(a)** g-factor spectral profiles of L-HNS synthesized at reaction durations of 5, 20, 40, 60, 120, and 240 minutes. The g-factor spectral profiles exhibit high consistency between synthesis durations of 120 and 240 minutes, demonstrating that the g-factor reaches a stable state after 120 minutes. (b) SEM images of L-HNS synthesized at 120 and 240 minutes exhibit consistent morphology, indicating that the synthesis process reaches a stable phase after 120 minutes.

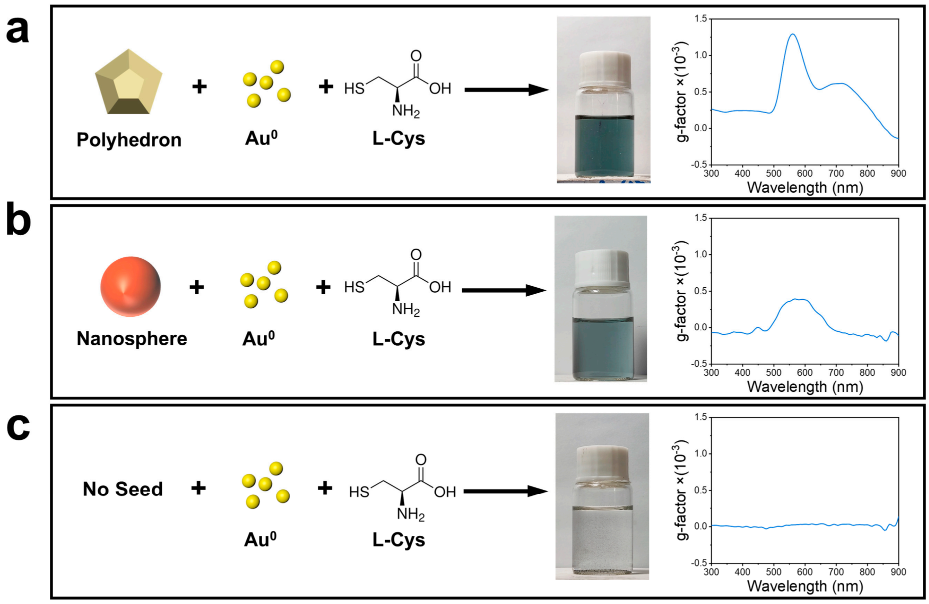

Figure S4. Comparative synthesis of gold nanostructures at different reaction systems. a-c) From left to right: synthetic process, optical image and g-factor spectrum of the obtained gold nanostructures by using gold polyhedrons as seeds (a), gold nanoparticles as seeds (b), and without any seeds (c), respectively. As demonstrated, the use of gold polyhedrons as seeds is essential for obtaining high concentration of chiral gold nanostructures.

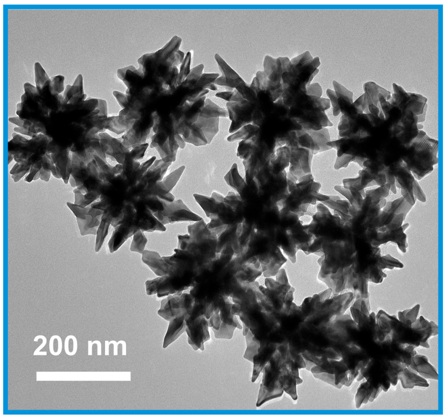

Figure S5. TEM image of L-HNS synthesized on gold polyhedrons by using 4 mM L-Cys as the chiral inducer.

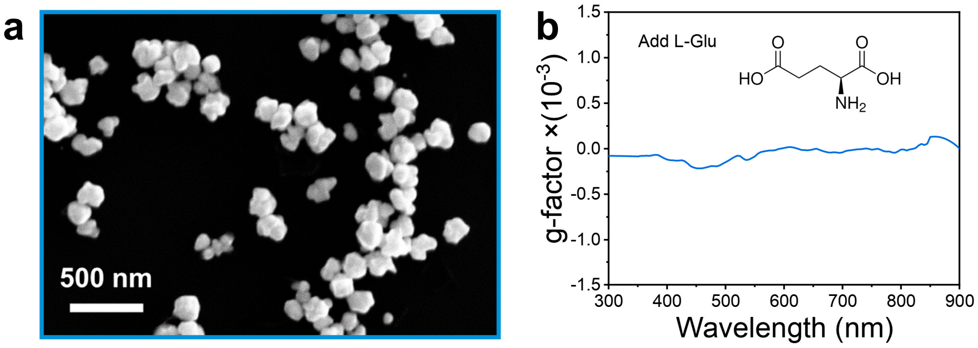

Figure S6. Characterizations of the gold nanostructures synthesized on gold polyhedrons by using 4 mM L-glutamic acid (L-Glu) as the chiral inducer: (a) SEM image and (b) g-factor spectrum. The

addition of L-Glu only produces irregularly shaped particles without any horns on them, and no significant chiral activity is observed for the product.

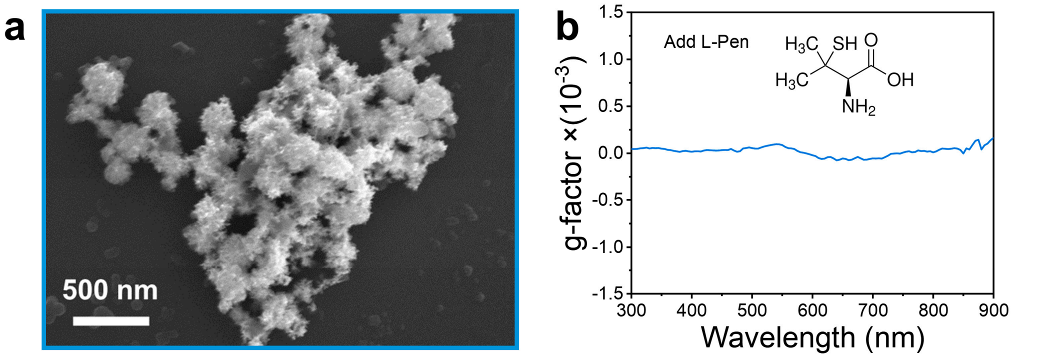

Figure S7. Characterizations of the gold nanostructures synthesized on gold polyhedrons by using 4 mM L-penicillamine (L-Pen) as the chiral inducer: (a) SEM image and (b) g-factor spectrum. The addition of L-Pen produces spiny nanostructure without chiral activity.

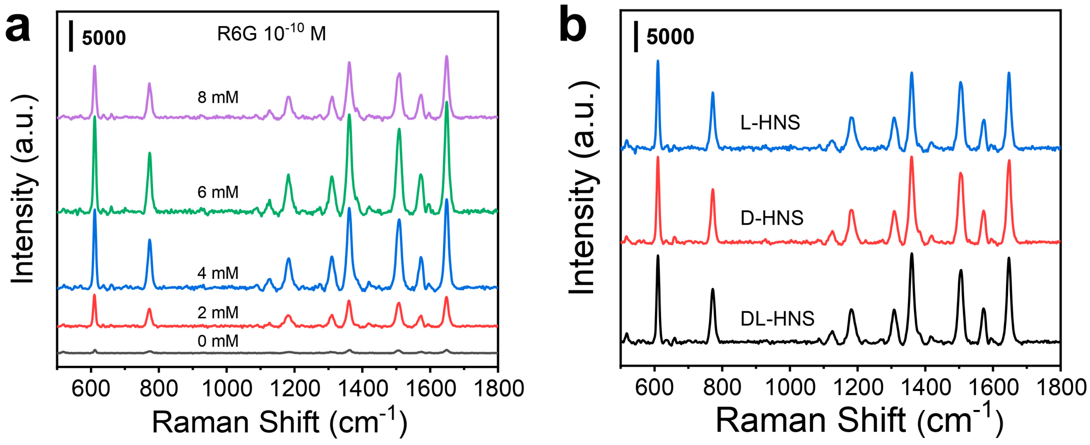

Figure S8. Comparison on SERS activity of varied gold nanostructures. (a) SERS spectra of 10^{-10} M R6G on L-HNS substrates synthesized by using 0, 2, 4, 6 and 8 mM L-Cys as chiral inducer. (b) SERS spectra of 10^{-10} M R6G on chiral and achiral HNS substrates synthesized under the induction of 4 mM L-Cys. The use of 6 mM L-Cys produces the gold nanostructures with the highest SERS ability, however, their chiral activity is much lower than the ones from 4 mM L-Cys. In the assistance of 4 mM cysteine (chiral and racemic ones), the obtained chiral and achiral HNSs exhibit almost the same SERS response to 10^{-10} M R6G.

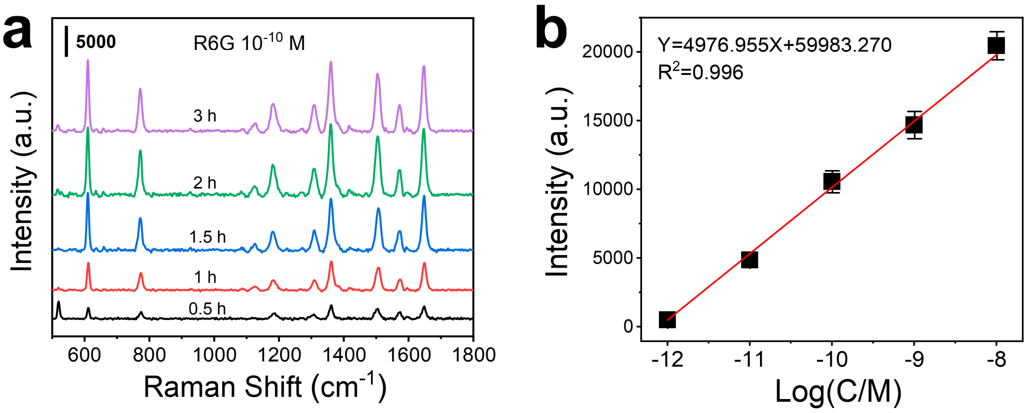

Figure S9. (a) Logarithmic scale linear fit of the Raman intensity of R6G at 610 cm^{-1} versus the concentration of R6G by using L-HNS as SERS substrate. (b) Linear fit of Raman intensity of R6G at 610 cm^{-1} versus the adsorption time on L-HNS substrates. Data are presented as mean ± SD. (n = 5).

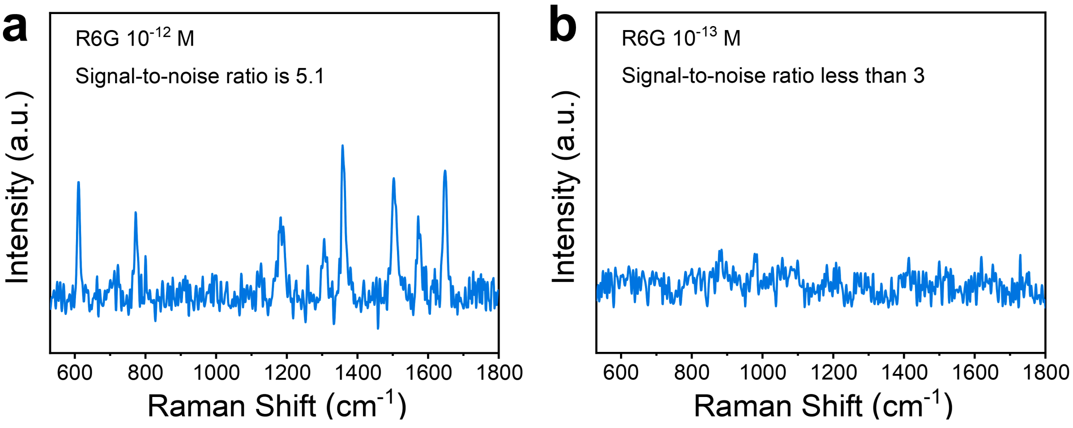

Figure S10. Detecting sensitivity of L-HNS to R6G molecules via enhancement of Raman signal. (a) SERS spectrum of 10^{-12} M R6G on L-HNS substrate with a signal-to-noise ratio of 5.1. (b) SERS spectrum of 10^{-13} M R6G on L-HNS substrate with a signal-to-noise ratio of < 3. Considering that the signal-to-noise ratio should be larger than 3 for a reliable signal, the sensitivity for R6G on L-HNS substrate is measured as 10^{-12} M.

Note S1: Calculation of Raman enhancement factor (EF)

As known, the Raman enhancement factor of a substrate can be calculated according to the following equation:

$$EF = \frac{\left(\frac{I_{SERS}}{N_{SERS}}\right)}{\left(\frac{I_{bulk}}{N_{bulk}}\right)} = \left(\frac{I_{SERS}}{I_{bulk}}\right) * \left(\frac{N_{bulk}}{N_{SERS}}\right) \qquad \text{(Equation} - 1\text{)}$$

where I_{SERS} and I_{bulk} are the Raman intensities of R6G adsorbed on SERS substrate and bulk R6G molecules, respectively, and N_{SERS} and N_{bulk} are the number of molecules involved in the SERS experiments and in the Raman measurement of bulk R6G, respectively. Here we use the Raman peak of R6G at 610 cm^{-1} to estimate the EFs.

Table S1. SERS intensity of R6G adsorbed on L-HNS and blank. Laser power: 2.5 mW, acquisition time: 10 s (n = 5).

Substrate	L-HNS	Blank
Concentration of R6G	10^{-12} M	10^{-2} M
Intensity (a.u.)	85.25	5746.94

Further, the N_{SERS} and N_{bulk} can be calculated by:

$$N_{\mathrm{SERS}} = \frac{cVN_{\mathrm{A}}\mathrm{A}_1}{\mathrm{A}_{\mathrm{sub}}} \quad \text{(Equation} - 2)$$

$$N_{\mathrm{bulk}} = \frac{\rho h N_{\mathrm{A}}\mathrm{A}_1}{\mathrm{M}} \quad \text{(Equation} - 3)$$

The $\frac{N_{\mathrm{bulk}}}{N_{\mathrm{SERS}}}$ can be derived as:

$$\frac{N_{\mathrm{bulk}}}{N_{\mathrm{SERS}}} = \frac{\rho h \mathrm{A}_{\mathrm{sub}}}{\mathrm{McV}} \quad \text{(Equation} - 4)$$

for N_{SERS}, c is the dye concentration, V is the dye volume on substrate, N_{A} is the Avogadro constant, A_1 is the laser spot area, and $\mathrm{A}_{\mathrm{sub}}$ is the substrate area. In the SERS experiments, the SERS substrates ($\mathrm{A}_{\mathrm{sub}} \approx 0.25\ \mathrm{cm}^2$) were soaked in R6G ethanol solution for 30 min, so we estimate the volume as 20 μL. For N_{bulk}, ρ is the density of bulk R6G (1.15 g/cm^3), $h = \frac{2\lambda}{N_{\mathrm{A}}{}^2}$ is the laser penetration depth with the λ of 532 nm and N_{A} (Numerical Aperture) of 0.4, and M is the molar mass of dye (479.02 g/mol for R6G). To estimate the N_{bulk} the excessive and high-concentration R6G ethanol solution (10^{-2} M) was coated onto the bare Si substrate, so the bulk R6G crystals can be formed after the solution was dried out. Hence, we can use the density of R6G to calculate the number of molecules. Taking all the above-mentioned factors into account, the EF in the SERS measurements can be calculated is 2.8×10^8.

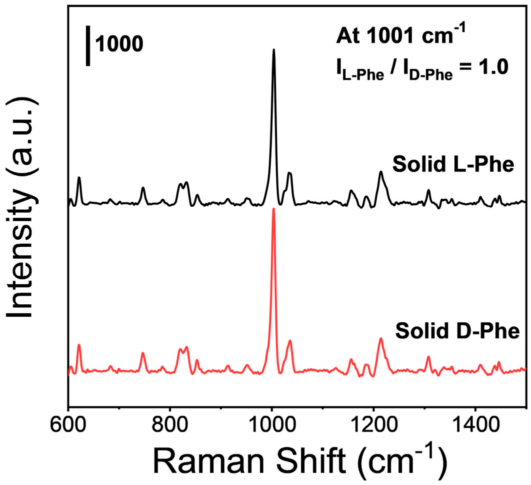

Figure S11. Raman spectra of solid L- and D-Phe on silicon wafer, which indicate that there is no observable differences in Raman intensity for solid L- and D-Phe.

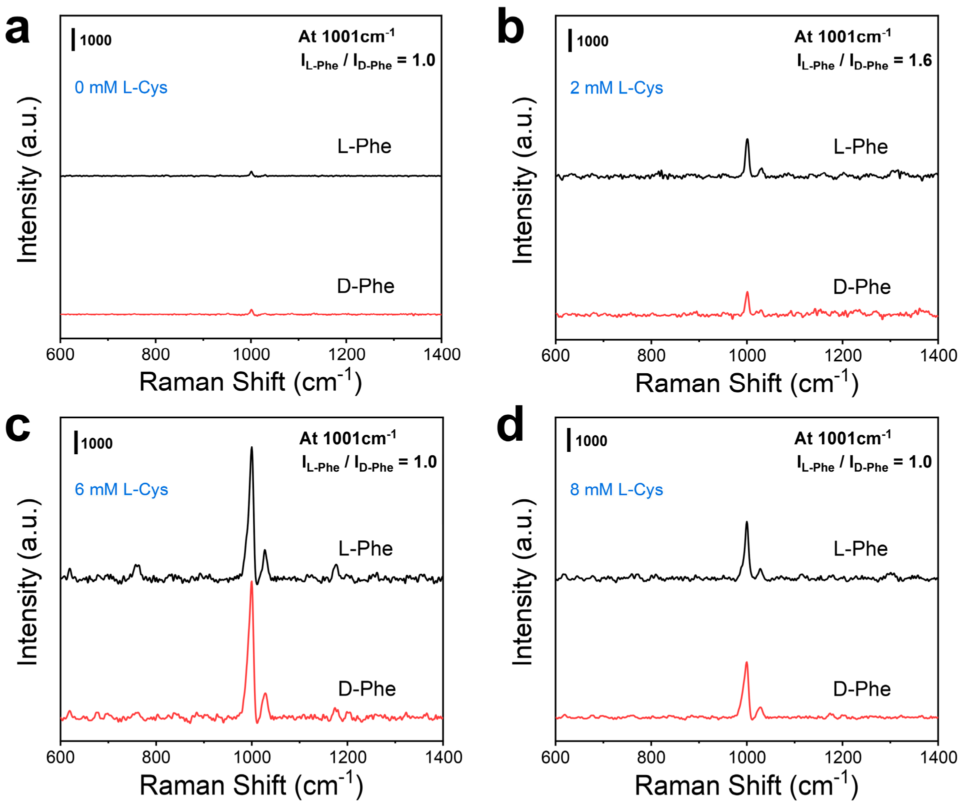

Figure S12. Comparison on SERS spectra of L- and D-Phe on varied HNS substrates, which are synthesized by using 0 (a), 2 (b), 6 (c) and 8 (d) mM L-Cys as chiral reducers, respectively. These HNS substrates are difficult to discriminate chiral Phe enantiomers, due to their poor SERS ability or low chiral activity. All data are the average of five replicates.

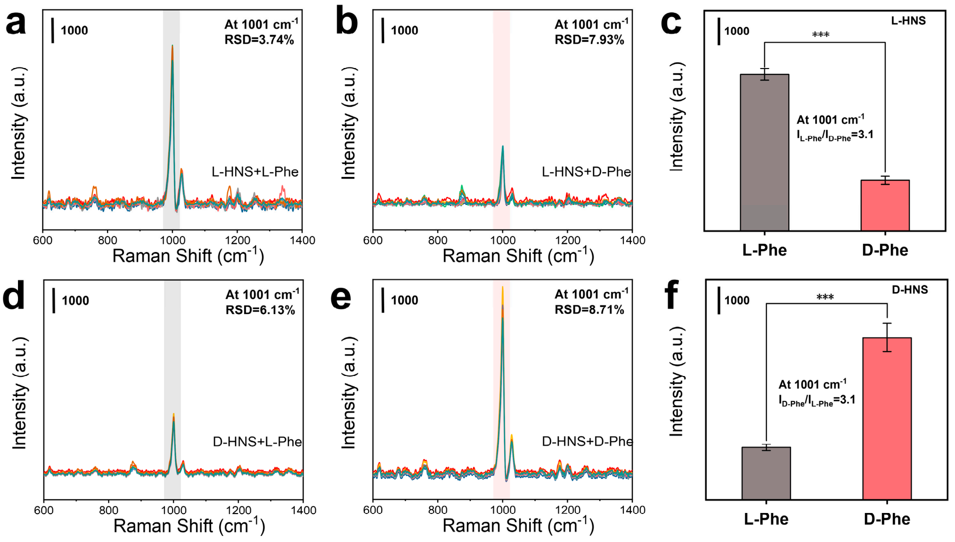

Figure S13. (a) and (b) show the Raman spectra of L-Phe and D-Phe (0.5 mM) measured on L-HNS substrates. Raman intensity of L-Phe at 1001 cm^{-1} (mean = 5688.75, SD =213.05, n = 10). Raman intensity of D-Phe at 1001 cm^{-1} (mean = 1835.08, SD = 145.52, n = 10). (c) The SERS intensity ratio of L-Phe and D-Phe at 1001 cm^{-1} on L-HNS. Data are presented as mean ± SD. (d) and (e) show the Raman spectra of L-Phe and D-Phe (0.5 mM) measured on D-HNS substrates. Raman intensity of L-Phe at 1001 cm^{-1} (mean = 1899.51, SD = 116.51, n = 10). Raman intensity of D-Phe at 1001 cm^{-1} (mean = 5901.18, SD = 513.95, n = 10) (f) The SERS intensity ratio of L-Phe and D-Phe at 1001 cm^{-1} on D-HNS. Data are presented as mean ± SD. *** $p < 0.001$.

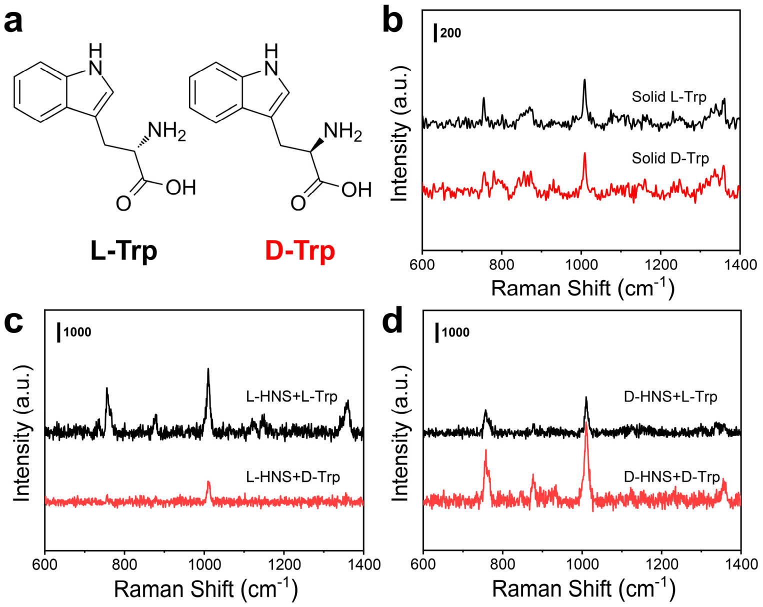

Figure S14. Differentiated SERS response of chiral HNSs to tryptophan (Trp) enantiomers. (a) Molecular structures of L-Trp and D-Trp. (b) SERS spectra of solid L-Trp and D-Trp on silicon wafer. (c) SERS spectra of 5 mM L- and D-Trp on L-HNS substrate. (d) SERS spectra of 5 mM L- and D-Trp on D-HNS substrate.

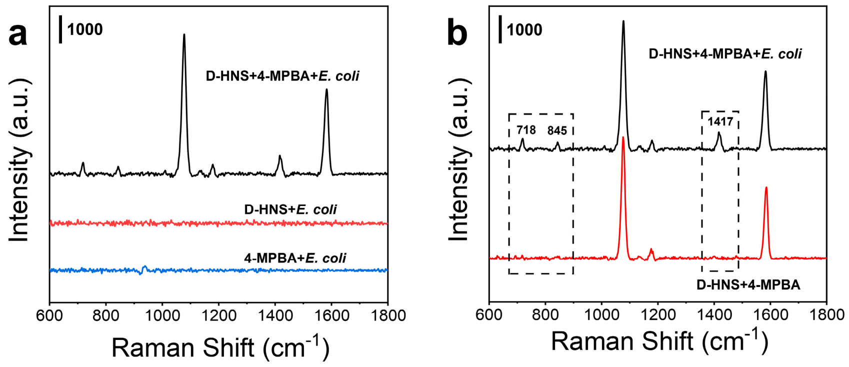

Figure S15. SERS detection of *E. coli* via using D-HNS as substrate and 4-MPBA as specific Raman probe. (a) SERS spectrum of mixed *E. coli* and 4-MPBA on D-HNS substrate, compared with the one from 4-MPBA. As seen, three new peaks at 718, 845, and 1417 cm^{-1} appear after introduction of *E. coli* into 4-MPBA solution. (b) SERS spectra of different material's systems: *E. coli* and 4-MPBA on silicon, *E. coli* on D-HNS substrate, and *E. coli* and 4-MPBA on D-HNS substrate. Via comparison, the Raman peak at 1417 cm^{-1} can be used as the SERS signal for *E. coli* on D-HNS substrate in the assistance of 4-MPBA.

Table S2. The tentative band assignment of SERS spectra of 4-MPBA-HNSs incubated with bacteria of *E. coli* according to the literatures.

Raman shift/cm^{-1}	Band assignments*
718	adenine, polyadenine, glycosidic ring mode (bacteria)
845	ring breathing Tyr protein (bacteria)
1076	βCCC + νCS (4-MPBA)
1181	βCH + βBOH (4-MPBA)
1417	ν_s(COO–) amino acids (bacteria)
1578	υCC (4-MPBA)

* Approximate description of the modes (υ, stretch; δ and γ, bend).

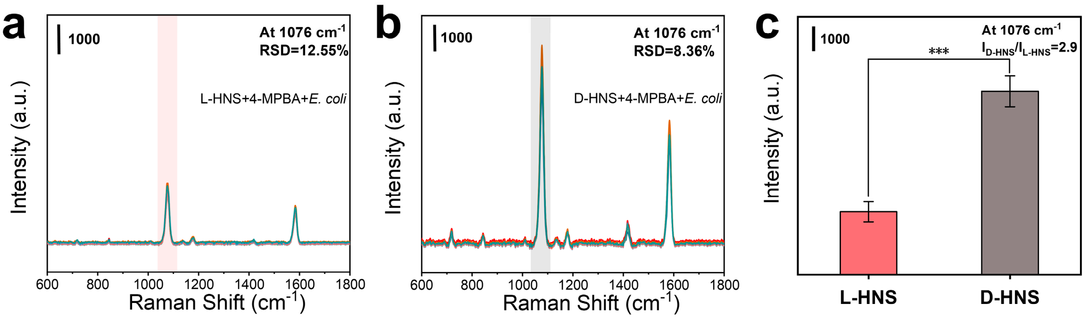

Figure S16. (a) and (b) showed Raman spectra for detecting *E. coli* on L-HNS and D-HNS substrates modified with 4-MPBA, respectively. Raman intensity of L-HNS substrate at 1076 cm^{-1} (mean = 1952.91, SD = 245.09, n = 10). Raman intensity of D-HNS substrate at 1076 cm^{-1} (mean = 5683.41, SD = 475.28, n = 10). (c) The SERS intensity ratio of *E. coli* at 1076 cm^{-1} on L-HNS and D-HNS substrates modified with 4-MPBA. Data are presented as mean ± SD. The error bar represents the SD. *** $p < 0.001$.

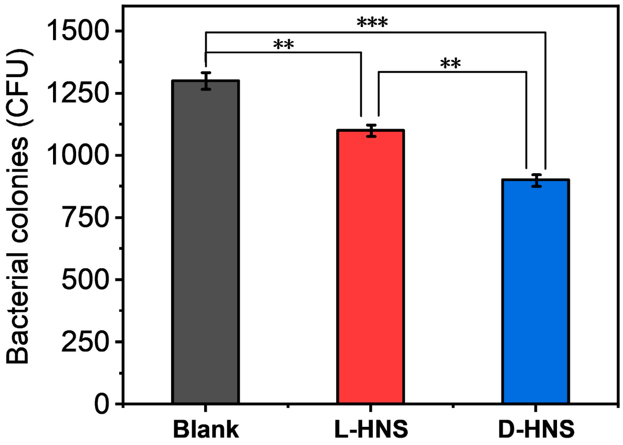

Figure S17. Count the bacterial colonies of *E. coli* without adding gold nanoparticles, with the addition of L-HNS and D-HNS. The data is expressed as mean ± SD. Blank: (mean = 1300, SD = 32.86, n = 5), L-HNS: (mean = 1100, SD = 22.80, n=5), D-HNS: (mean = 900, SD = 23.87, n=5). The error bar represents the SD. *$p < 0.01$, **$p < 0.01$, ***$p < 0.001$.

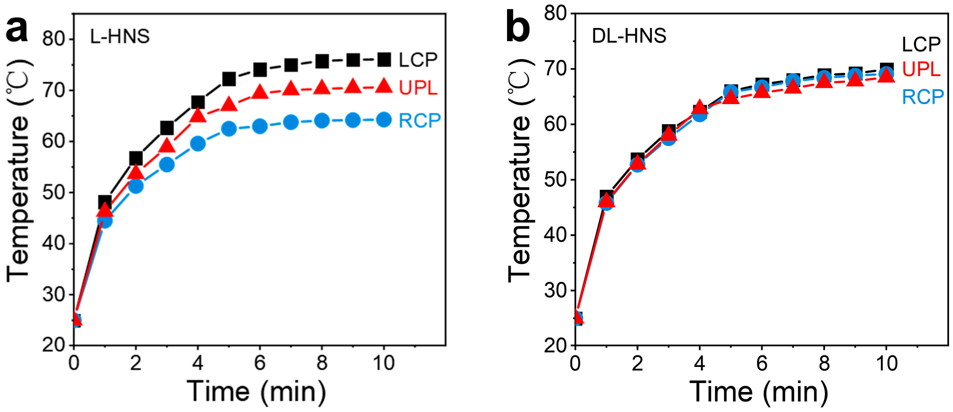

Figure S18. (a) Temperature-rising curves of L-HNS solution with irradiation time under LCP, RCP and UPL light (2.5 W/cm^2). (b) Temperature-rising curves of DL-HNS solution under LCP, DCP and UPL laser irradiation (2.5 W/cm^2). Interestingly, chiral HNS exhibits a different photothermal response to circularly polarized light. All experiments were repeated five times, and one representative set of data was selected for plotting.

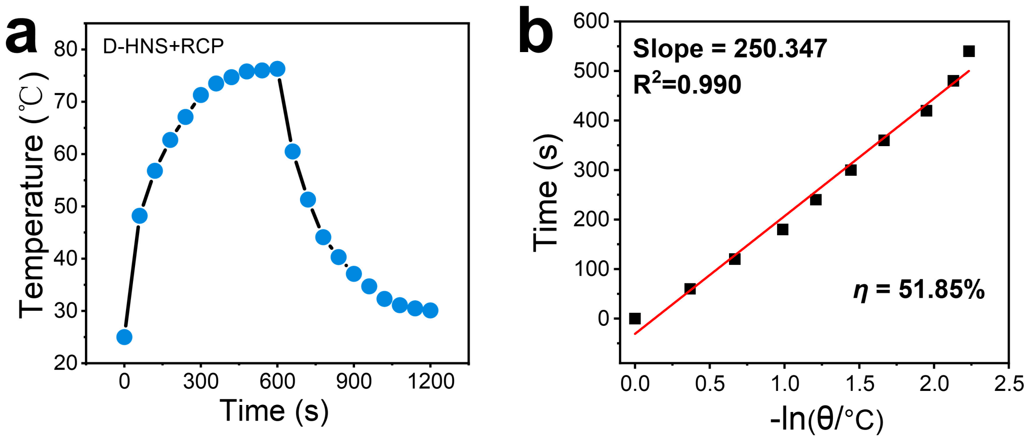

Figure S19. (a) Temperature profile of D-HNS under photothermal RCP heating and natural cooling (2.5 W/cm^2). (b) Calculation of time constant for heat transfer using linear regression cooling profile. $\theta = (T - T_S)/(T_{max} - T_S)$. $T_S$is the ambient temperature; T_{max} is the equilibrium temperature before turning off the laser. All experiments were repeated five times, and one representative set of data was selected for plotting.

Note S2: Photothermal performance of D-HNS under RCP.

The heat transfer time constant (τ) was calculated based on the heating-cooling curve using linear regression analysis of the cooling curve, and the photothermal conversion efficiency (η) was determined to be 51.85%, as shown below:

$$\eta = \frac{[hS(T_{max} - T_{surr}) - Q_{dis}]}{I(1 - 10^{-A808})} \qquad \text{(Equation} - 5)$$

$$\tau s = \frac{m_D C_D}{hS} \qquad \text{(Equation} - 6)$$

where the equilibrium maximum temperature (T_{max}= 76.5℃) and the ambient temperature (T_{surr} = 25.0℃) are obtained. Further, I is the laser power (2.5 W/m^2) and A808 is the absorbance at 808 nm, which is 2.0. Furthermore, where h and S (m^2) respectively denote the heat transfer coefficient and the surface area of the container, and hS can be calculated from Equation 2 in which τ is (250.3 s) and m_D and C_D are the mass (1.5 g) and the heat capacity (4.2 J/g) of deionized water, respectively. Q_{dis} can be measured independently to be 14.3 mW by using deionized water under the other identical conditions.

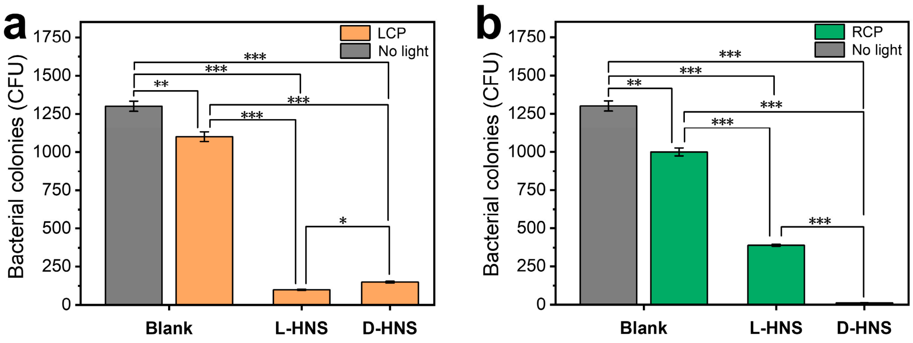

Figure S20. (a) The colony count of *E. coli* in the blank group without light source, LCP blank group, and after treatment with LCP and L/D-HNS combination. The data is expressed as mean ± SD. Blank with no light: (mean = 1300, SD = 32.86, n = 5), Blank with LCP: (mean = 1100, SD = 31.80, n = 5), L-HNS with LCP: (mean = 100, SD = 3.61, n = 5), D-HNS with LCP: (mean = 150, SD = 5.87, n = 5). The error bar represents the SD. (b) The colony count of *E. coli* in the blank group without light source, RCP blank group, and after treatment with RCP and L/D-HNS combination. The data is expressed as mean ± SD. Blank with no light: (mean = 1300, SD = 35.72, n = 5), Blank with RCP: (mean = 1000, SD = 25.87, n = 5), L-HNS with RCP: (mean = 390, SD = 6.23, n = 5), D-HNS with RCP: (mean = 11, SD = 1.10, n = 5). The error bar represents the SD. *$p < 0.01$, **$p < 0.01$, ***$p < 0.001$.
